# Supplementary material for: EnzML: multi-label prediction of enzyme classes using InterPro signatures
Source: BMC Bioinformatics. 2012 Apr 25;13:61. doi: 10.1186/1471-2105-13-61 (PMC3483700; doi:10.1186/1471-2105-13-61)
Supplement: Addtional file 5 — The Java code to format the data files, evaluate and predict. The file enzml_java_code.tar.gz contains the Java code used to format database data to ARFF and XML formats, to execute cross and train-test (jackknife) evaluations and to record evaluation results to database. More information is included in the readme.txt file and the Javadoc files. The code can be used with a MySQL database. To use a different database software, other JDBC drivers might be required. [file 1471-2105-13-61-S5.gz › java_code/enzml2011/doc/uk/ac/ed/inf/enzml/test/dataharness/package-summary.html]

uk.ac.ed.inf.enzml.test.dataharness


---


|  |  |  |
| --- | --- | --- |
| **Overview** \| **Package** \| Class \| **Use** \| **Tree** \| **Deprecated** \| **Index** \| **Help** | |  |
| **PREV PACKAGE**   **NEXT PACKAGE** | **FRAMES**    **NO FRAMES**     **All Classes** |  |


---

## Package uk.ac.ed.inf.enzml.test.dataharness

| **Class Summary** | |
| --- | --- |
| **AllDataTests** | Data harness for arff file generation and learning tests. |
| **ArffPropsFilesTest** | Test the ARFF properties files used in the test harness and their content. |
| **ArffPropsOneTest** | Get the arff data properties from file |
| **ArffPropsQueriesOneTest** | Class |
| **ArffPropsQueriesTest** | Checks the results of the queries contained in the arff properties files |
| **ArffPropsQueriesTwoTest** | Class |
| **ArffPropsTwoTest** | Class |
| **CreateDataTable** | Create a table for test instances (and their attributes and classes) |
| **DatabaseTest** | Check the test database |
| **DataOne** | Class |
| **DataTableOneTest** | Basic data table containing 3 columns with: instances, attributes, classes. |
| **DataTableTwoTest** |  |
| **DataTwo** | Class |
| **PathTest** |  |
| **PathTestTest** | Class |

---


|  |  |  |
| --- | --- | --- |
| **Overview** \| **Package** \| Class \| **Use** \| **Tree** \| **Deprecated** \| **Index** \| **Help** | |  |
| **PREV PACKAGE**   **NEXT PACKAGE** | **FRAMES**    **NO FRAMES**     **All Classes** |  |


---
